# Microbiota signatures associated with invasive *Candida albicans* infection in the gastrointestinal tract of immunodeficient mice

**DOI:** 10.3389/fcimb.2023.1278600

**Published:** 2024-01-17

**Authors:** Jia-Ying Yan, Tsung-Han Lin, Yu-Tang Jong, Jun-Wei Hsueh, Sze-Hsien Wu, Hsiu-Jung Lo, Yee-Chun Chen, Chien-Hsiung Pan

**Affiliations:** ^1^ National Institute of Infectious Disease and Vaccinology, National Health Research Institutes, Miaoli, Taiwan; ^2^ School of Dentistry, China Medical University, Taichung, Taiwan; ^3^ Department of Medicine, National Taiwan University, Taipei, Taiwan; ^4^ Graduate Institute of Biomedical Sciences, China Medical University, Taichung, Taiwan; ^5^ Graduate Institute of Medicine, Kaohsiung Medical University, Kaohsiung, Taiwan

**Keywords:** *Candida albicans*, microbiota signature, immunodeficient mice, invasive candidiasis, biomarker, *Bacteroides vulgatus*, *Candidatus Arthromitus*, IL-22

## Abstract

*Candida albicans* is a commensal microorganism in the human gut but occasionally causes invasive *C. albicans* infection (ICA), especially in immunocompromised individuals. Early initiation of antifungal therapy is associated with reduced mortality of ICA, but rapid diagnosis remains a challenge. The ICA-associated changes in the gut microbiota can be used as diagnostic and therapeutic targets but have been poorly investigated. In this study, we utilized an immunodeficient Rag2γc (*Rag2*-/-*il2γc*-/-) mouse model to investigate the gut microbiota alterations caused by *C. albicans* throughout its cycle, from its introduction into the gastrointestinal tract to invasion, in the absence of antibiotics. We observed a significant increase in the abundance of *Firmicutes*, particularly *Lachnospiraceae* and *Ruminococcaceae*, as well as a significant decrease in the abundance of *Candidatus Arthromitus* in mice exposed to either the wild-type SC5314 strain or the filamentation-defective mutant (*cph1/cph1 efg1/efg1*) HLC54 strain of *C. albicans*. However, only the SC5314-infected mice developed ICA. A linear discriminate analysis of the temporal changes in the gut bacterial composition revealed *Bacteroides vulgatus* as a discriminative biomarker associated with SC5314-infected mice with ICA. Additionally, a positive correlation between the *B. vulgatus* abundance and fungal load was found, and the negative correlation between the *Candidatus Arthromitus* abundance and fungal load after exposure to *C. albicans* suggested that *C. albicans* might affect the differentiation of intestinal Th17 cells. Our findings reveal the influence of pathogenic *C. albicans* on the gut microbiota and identify the abundance of *B. vulgatus* as a microbiota signature associated with ICA in an immunodeficient mouse model.

## Introduction

*Candida albicans* is a commensal organism in the human gastrointestinal (GI) tract but can cause opportunistic infections when the host immune system or gut microbiota is disrupted (Gow et al., 2012; Iliev et al., 2012). Invasive candidiasis, mainly caused by 5 pathogens: *C. albicans*, *Nakaseomyces glabrata* (previously known as *C. glabrata*), *C. tropicalis*, *C. parapsilosis* and *Pichia kudriavzevii* (previously known as *C. krusei*) (McCarty et al., 2021), is the most prevalent fungal disease in humans and has mortality rates reaching 40% or more despite early initiation of antifungal therapy (Chen et al., 2014; Conti et al., 2015; McCarty et al., 2021). The risk factors for invasive candidiasis includes broad-spectrum antibiotics, abdominal surgery, immunosuppressants, central venous catheter and long-term stay in intensive care unit (Pappas et al., 2018). Invasive *C. albicans* infection (ICA) is responsible for more than 50% of invasive candidiasis cases worldwide, even the infections caused by non-*albicans* species are increasing (Guinea, 2014). It is believed that endogenous commensal *C. albicans* in the GI tract is responsible for ICA (Voss et al., 1994; Marco et al., 1999). The lack of specific biomarkers to distinguish between commensal and pathogenic *C. albicans* hinders the diagnosis of ICA (Leon et al., 2014). Nonculture-based diagnostic tests targeting *C. albicans*-specific molecules or antibodies are being developed to improve the low sensitivity of conventional diagnostic methods (Morrell et al., 2005; Authier et al., 2008), whereas the sensitivity of *C. albicans*-specific immunoassays in immunocompromised patients remains limited (Diez et al., 2021). Given the growing population of immunocompromised hosts and the emergence of *Candida* species as important pathogens of sepsis and healthcare-associated infections in the last three decades (Chen et al., 2014; Guinea, 2014; Conti et al., 2015; Jaeger et al., 2015), it is important to identify alternative diagnostic biomarkers for ICA other than immune-related molecules in immunocompromised individuals.

Microbiota signatures associated with specific pathogens or disorders have recently shown potential as diagnostic biomarkers (d'Enfert et al., 2021; Mizutani et al., 2021). Studies have reported that candidemia patients experience marked intestinal expansion of pathogenic *Candida*, which is associated with substantial decreases in the bacterial burden and diversity (Zhai et al., 2020). These findings suggest that microbiota changes could be used as diagnostic biomarkers for ICA. Gut bacteria play an important role in preventing *C. albicans* invasion or colonization in the gut (Phillips and Balish, 1966; Clark, 1971). However, little is known about the impact of pathogenic *C. albicans* on the gut microbiota, especially regarding the microbiota changes associated with invasive diseases (d'Enfert et al., 2021). The overgrowth of *C. albicans* can alter the microbial community via direct competition with gut bacteria for niches or nutrients (Mason et al., 2012b) or the secretion of metabolites that influence bacterial activity (McAlester et al., 2008). Alternatively, *C. albicans* can indirectly modulate bacterial communities by activating host immune responses (van Tilburg Bernardes et al., 2020). Even after decades of effort, the study of the interactions between *C. albicans* and gut bacteria remains challenging due to the colonization resistance exerted by gut bacteria, and antibiotic pretreatment is needed to disrupt the gut microbiota for successful *C. albicans* colonization in adult mice (Cole et al., 1996; Koh et al., 2008). Although germ-free mice transplanted with *C. albicans* and selected bacteria have been used to study the interactions between *C. albicans* and anaerobic bacteria (Balish et al., 1993; Eckstein et al., 2020), the simplified environment in germ-free mice differs from the complex community present in an intact gut microbiome. Therefore, studies investigating the gut microbiota changes caused by GI *C. albicans* infection have been limited and conducted in the context of antibiotic use.

We previously demonstrated successful colonization of *C. albicans* in the gut without antibiotic pretreatment using a severe immunodeficient mouse (*Rag2* ^-/-^ *il2γc* ^-/-^; Rag2γc), where indigenous *C. albicans* invasion is observed within 2-3 weeks after infection (Pan et al., 2020). The high susceptibility of Rag2γc mice to *C. albicans* has been attributed to the impaired IL-17A and IL-22 response due to the absence of functional T, B, and NK cells (Colucci et al., 1999), including type-3 innate lymphoid cells (Van Maele et al., 2014). In addition, pretreatment with antibiotics enhances the severity of invasive infection in *C. albicans*-infected Rag2γc mice, suggesting the protective role of the gut microbiota. However, the interaction between the gut microbiota and *C. albicans*, including the impacts of *C. albicans* on the gut bacteria and vice versa, in this immunodeficient mouse model is unclear.

Although dysbiosis of the gut microbiota is important for triggering the transition of *C. albicans* from commensal to pathogenic, in this study, we focused on the effect of pathogenic *C. albicans* after its introduction and invasion on the composition of the gut bacteria in susceptible Rag2γc mice by 16S rRNA gene sequencing and compared the differential microbiota alterations associated with ICA by infecting Rag2γc mice with wild-type SC5314 or filamentation-defective mutant HLC54 (*cph1/cph1 efg1/efg1*) *C. albicans*. The major differences in the microbiota changes between SC5314- and HLC54-infected mice were noted coincidently with the development of ICA, which was observed only in SC5314-infected mice. The results from a metagenomic analysis indicated that *Bacteroides vulgatus* was the major discriminative biomarker associated with SC5314-infected mice with ICA. Although our findings come from a single immunocompromised murine model and need further evaluation in clinical specimens, the understandings for the microbiota changes caused by *C. albicans* invasion provides an alternative strategy to accelerate the diagnosis of ICA and potentially reduce the mortality of immuno-compromised patients with ICA.

## Results

### Antibiotics are not required for *Candida albicans* to colonize the gut of immunodeficient Rag2γc mice and cause invasive candidiasis

**Figure 1:**
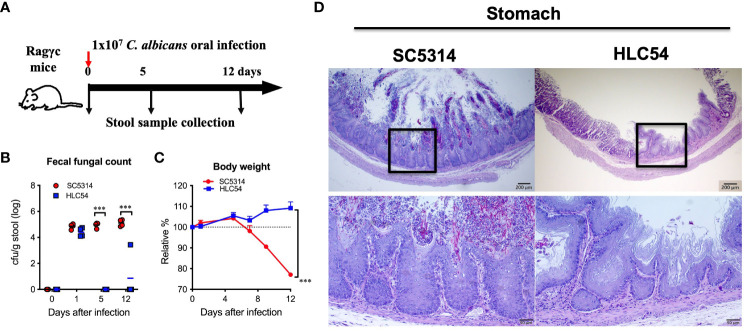
Severe gastritis caused by the invasion of wild-type *Candida albicans* but not filamentation-defective mutant in immunodeficient mice. A schematic diagram of the experimental design and sample collection schedule is shown in **(A)**. In brief, groups of immunodeficient Rag2γc mice (n=7) were orally injected with 1 x 10^7^ wild-type SC5314 or mutant HLC54 *C albicans* by gavage, and stool samples from individual mice were collected before infection and on days 5 and 12 post-infection. The fecal fungal counts determined by culture **(B)** and body weight changes **(C)** were recorded. Infected mice were sacrificed 12 days post-infection, and the stomach tissues were harvested for histological examination by PAS staining with hematoxylin counterstaining **(D)**. A typical image of SC5314- and HLC54-infected mice is shown, and the magnification of the black square area is shown at the bottom. All data were analyzed by 2-way ANOVA, and p values lower than 0.001 are indicated with ***.

To investigate the gut bacterial changes caused by *C. albicans* infection, we designed animal experiments according to previous reports (Pan et al., 2020) and collected stool samples before infection and at 5 and 12 days post-infection (to represent the time of introduction and invasion, respectively) for bacterial 16S rRNA gene sequencing (**Figure 1A**). The colonization of *C. albicans* in the gut was confirmed by measuring the fungal counts in the feces. Prior to infection, no fungus was detected in fecal cultures, and similar fungal counts were observed in mice infected with either wild-type SC5314 or hyphae-deficient HLC54 one day after infection (**Figure 1B**). However, the fecal fungal counts in HLC54-infected mice decreased rapidly, and only a few colonies were occasionally detected 12 days later. In contrast, the fecal fungal counts in SC5314-infected mice were maintained at high levels (*p*<0.001; n=7). Consistent with the higher fungal counts, severe body weight loss was observed one week later in SC5314-infected mice but not in HLC54-infected mice (**Figure 1C**). In addition, obvious tissue damage, including epithelial hyperplasia and erosion accompanied by hyphal fungi, was found in the stomach of SC5314-infected mice, but neither hyperplasia nor tissue-associated fungi were observed in HLC54-infected mice (**Figure 1D**). Our data suggest that SC5314- but not HLC54-infected Rag2γc mice developed gastric candidiasis at 12 days post-infection, similar to our previous findings, which revealed that SC5314-infected Rag2γc mice exhibit signs of invasive infection, with 58% positive results in kidney/liver cultures within 2-3 weeks post-infection (Pan et al., 2020).

### *C. albicans* colonization induced a temporary increase in the fecal microbiota diversity

**Figure 2:**
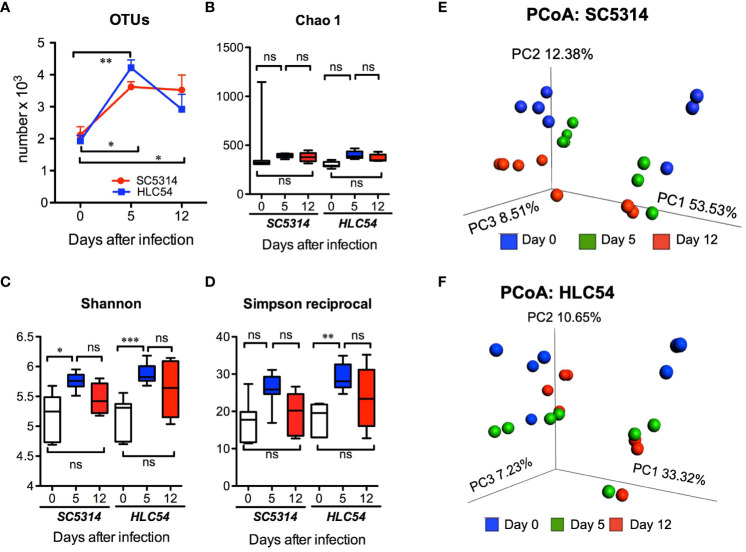
The alpha and beta diversity of the gut microbiota were enhanced in immunodeficient mice after *C. albicans* infection. The composition of the gut microbiota in Rag2γc mice (n=7) before infection (day 0) or at 5 and 12 days after either wild-type SC5314 or filamentation-defective mutant HLC54 infection was analyzed by 16S rRNA gene sequencing. The alpha diversity described by the number of OTUs **(A)** and the Chao 1 **(B)**, Shannon **(C)** and Simpson reciprocal indices **(D)** is shown. The beta diversity within the microbial communities of SC5314- **(E)** or HLC54-infected **(F)** mice was further assessed by conducting principal coordinate analysis (PCoA) based on weighted UniFrac distances. All data were analyzed by 2-way ANOVA, and p values lower than 0.05, 0.01 and 0.001 are indicated with *, ** and ***, respectively. ("ns" indicates no significance).

To understand the influence of *C. albicans* on gut bacteria, we performed a longitudinal analysis by collecting fresh stool samples from individual mice (n=7, combined data from two independent experiments) at particular times for 16S rRNA sequencing. We first examined the species richness, evenness and biodiversity of the gut microbiota by measuring the alpha diversity. Compared to the pre-infection (day 0) level, the number of operational taxonomic units (OTUs) was significantly increased after exposure to HLC54 or SC5314 (*p*<0.01 and *p*<0.05, respectively; **Figure 2A**). However, 12 days later, the number of OTUs decreased to levels comparable to those noted at the pre-infection phase in HLC54-infected mice but not in SC5314-infected mice, which still showed a significantly higher OTU number than that seen before infection (*p*<0.05). Next, we determined the alpha diversity by calculating the Chao 1, Shannon and Simpson indices with QIIME. No significant difference was observed in the Chao 1 index (**Figure 2B**); however, the Shannon index, which measures the gut bacterial complexity, was increased significantly on day 5 in both SC5314- and HLC54-infected mice (*p*<0.05 and *p*<0.001 compared to the pre-infection stage, respectively) and was restored to the pre-infection level on day 12 (**Figure 2C**). The Simpson index, which refers to the evenness of the gut bacterial community, increased after exposure to *C. albicans* in both SC5314- and HLC54-infected mice, but only the latter mice showed significant differences from their pre-infection levels (*p*<0.001; **Figure 2D**). Parallel to the Shannon index, the Simpson index returned to the pre-infection levels on day 12 in both SC5314- and HLC54-infected mice. All of these indices indicated a clear trend in which the gut microbiota alpha diversity increased temporarily after exposure to *C. albicans* and returned to the pre-infection levels on day 12. In contrast to alpha diversity, which measures the diversity of bacteria within a sample, beta diversity describes the distance and dissimilarity between individual samples. We performed principal coordinate analysis (PCoA) and found that the individual samples within the same group were separated according to the two independent experiments (n=4 and n=3) and further separated based on the time since infection, i.e., pre-infection and 5 and 12 days post-infection (**Figure 2E**). The same trends found for the positions of the groups corresponding to different times between the two independent experiments suggested that the intergroup changes were consistent. The major difference between SC5314- and HLC54-infected mice was that the gut composition of HLC54-infected mice on days 5 and 12 clustered more closely together than that of SC5314-infected mice (**Figure 2F**). These results suggested that the difference in the bacterial composition of HLC54-infected mice between days 5 and 12 was smaller than that found for the bacterial composition of SC5314-infected mice.

### *C. albicans* colonization induced an increase in the abundance of *Firmicutes* but a decrease in the abundance of *Bacteroidetes* and *Verrucomicrobia*

**Figure 3:**
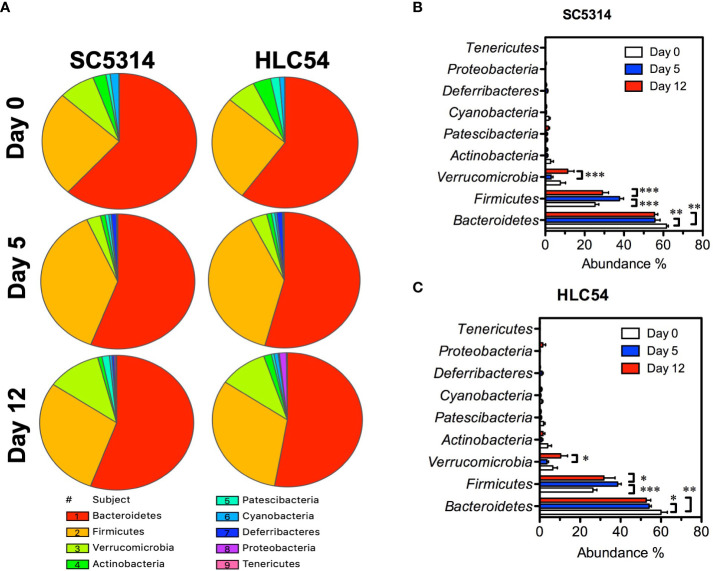
Phylum-level analysis of the gut microbiota alterations caused by wild-type and avirulent *Candida albicans*. The relative abundance of the gut bacterial taxa at the phylum level in Rag2γc mice (n=7) before infection (day 0) or at 5 and 12 days after *C. albicans* infection is represented with a pie chart that was created using SPICE 6 software **(A)**. The mean and standard deviation of the abundance of the gut bacterial phyla in SC5314-infected **(B)** or HLC54-infected **(C)** mice are shown. A 2-way ANOVA was used for analysis, and p values lower than 0.05, 0.01 and 0.001 are indicated with *, ** and ***, respectively.

To understand the changes in the composition of gut microbiota, we assessed the taxonomic profiles of bacteria by 16S rRNA sequencing. The taxonomic analysis revealed that *Bacteroidetes*, *Firmicutes* and *Verrucomicrobia* were the predominant phyla in both SC5314- and HLC54-infected mice (**Figure 3A**), in line with the results from studies of other immunodeficient mice (Van Averbeke et al., 2022). Both SC5314 and HLC54 infections caused gut microbiota dysbiosis by increasing the *Firmicutes* abundance and reducing the *Bacteroidetes* and *Verrucomicrobia* abundance (**Figures 3B, C**). The *Firmicutes* abundance significantly increased on day 5 and was restored to pre-infection levels on day 12. Conversely, the *Verrucomicrobia* abundance temporally decreased and returned to pre-infection levels on day 12. In contrast to the short-term changes in the *Firmicutes* and *Verrucomicrobia* abundance, the *Bacteroidetes* abundance was persistently and significantly decreased after exposure to SC5314 or HLC54 on days 5 and 12 compared to the pre-infection levels. Although the SC5314-infected mice had higher fungal counts in feces and developed severe tissue lesions, the changes in the gut microbiota between SC5314- and HLC54-infected mice were quite similar at the phylum level.

### Differences in the fecal microbiota between SC5314- and HLC54-infected mice were detected on day 12 post-infection

**Figure 4:**
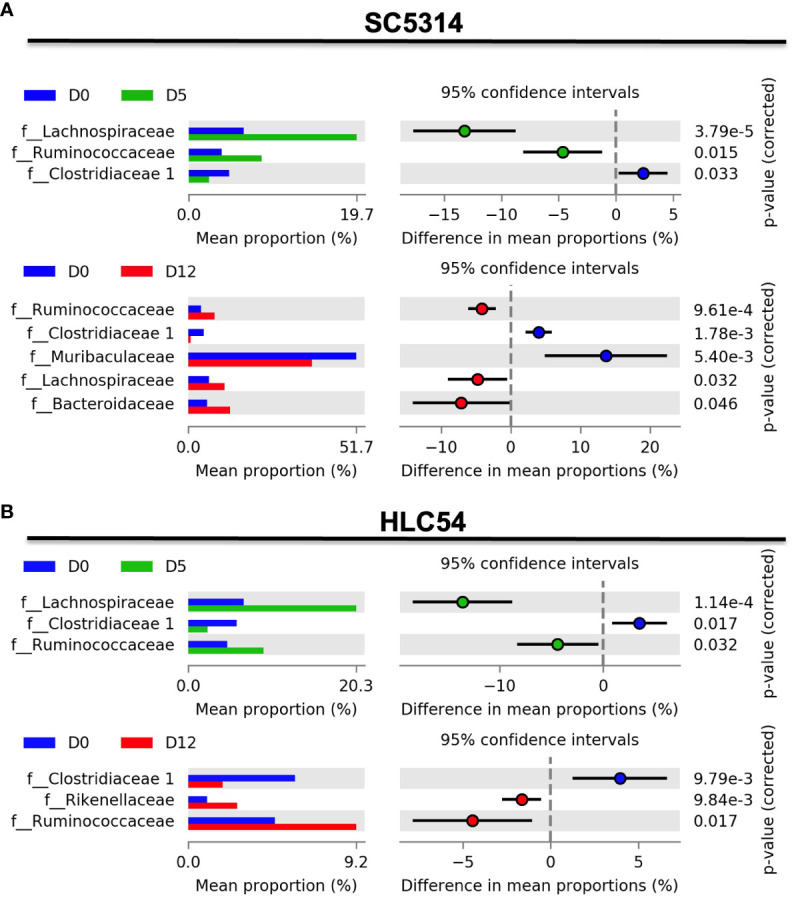
The time-dependent differential gut microbiota changes at the family level were observed after *C. albicans* infection. Family-level comparisons of the gut microbiota between pre-infection (D0) and 5 days (D5) post-infection or between pre-infection (D0) and 12 days (D12) post-infection in Rag2γc mice (n=7) infected with either SC5314 **(A)** or HLC54 **(B)** were performed by STAMP analysis. The left panel represents the mean proportion of the relative abundance of differential bacterial families, and the right panel shows the statistical results, including the differences in the mean proportion between 2 groups, 95% confidence intervals and corrected p values.

To further clarify the microbiota changes after exposure to SC5314 and HLC54, we subsequently analyzed the differences in the abundance of gut bacteria using Statistical Analysis of Metagenomic Profiles (STAMP) software. Among the abundant families (with a relative abundance ≥1%) of the *Firmicutes* phylum, a significant increase in the abundance of *Lachnospiraceae* and *Ruminococcaceae* and a significant decrease in the abundance of *Clostridiaceae* I were found on days 5 and 12 after SC5314 infection compared with the pre-infection level (**Figure 4A**). Within the *Bacteroidetes* phylum, no family showed any significant difference in abundance on day 5, but two families displayed substantial alterations on day 12: an increase in the *Bacteroidaceae* abundance and a decrease in the *Muribaculaceae* abundance (*p*<0.05 and *p<0.01* vs. the pre-infection levels, respectively). HLC54-infected mice showed the same gut microbiota changes on day 5 but minor alterations on day 12 compared with SC5314-infected mice (**Figure 4B**). These families with significant changes in abundance included *Clostridiaceae* I and *Ruminococcaceae*, which showed the same changes between SC5314- and HLC54-infected mice, as well as a unique change in *Rikenellaceae* (within the *Bacteroidetes* phylum), which displayed a significant increase in abundance (*p<0.01*, compared to the pre-infection level). These data suggested that HLC54-infected mice displayed the same gut microbiota alterations as SC5314-infected mice on day 5 but minor disturbances compared with SC5314-infected mice on day 12 post-infection.

### An increase in the *Bacteroides vulgatus* abundance was detected in SC5314- but not HLC54-infected mice

**Figure 5:**
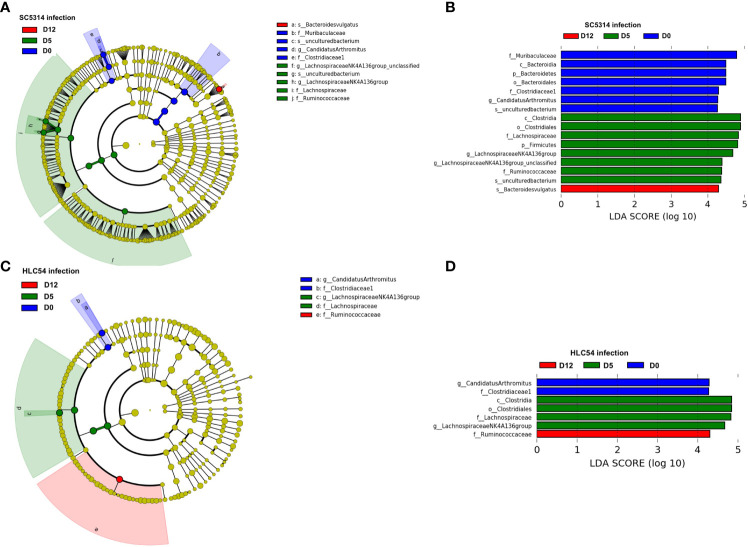
Identification of discriminative biomarkers associated with *Candida albicans* invasion. Longitudinal comparisons of the gut microbiota between pre-infection (D0) and 5 (D5) or 12 days (D12) post-infection of Rag2γc mice (n=7) infected with either *C. albicans* SC5314 or HLC54 by LEfSe analysis **(A)** and **(C)** or thresholding based on an LDA score ≥ 4.0 **(B)** and **(D)**. The cladogram results were calculated by LEfSe and depicted, and the discriminative biomarkers for each group are represented by different colors. The effect size of each taxon was evaluated by the Wilcoxon sum-rank test followed by linear discriminant analysis.

To identify the specific bacterial taxa associated with *C. albicans* invasion, we compared the time-dependent changes in the gut microbiota composition after infection by linear discriminate analysis effect size (LEfSe) analysis (Segata et al., 2011). A cladogram shows the structure of the gut microbiota and the differentially abundant bacteria before infection and 5 and 12 days after exposure to *C. albicans*. In agreement with the results from the STAMP analysis, most of the alterations occurred within the *Lachnospiraceae* and *Ruminococcaceae* families 5 days post-infection (**Figure 5A**). At the genus level, LEfSe analysis revealed the *Candidatus Arthromitus* and *Lachnospiraceae NK4A136* groups as biomarkers that discriminate between pre-infection and 5 days post-infection according to a linear discriminate analysis (LDA) score ≥4 (**Figure 5B**). For the time of *C. albicans* invasion (on day 12), the species *Bacteroides vulgatus* was the only discriminative biomarker based on the same cutoff LDA score. A cladogram showed that the structure of the gut microbiota of HLC54-infected mice was different from that of SC5313-infected mice, mainly on day 12 (**Figure 5C**). The same biomarkers discriminating between pre-infection and 5 days post-infection (*Candidatus Arthromitus* and *Lachnospiraceae NK4A136* group, respectively) were found between SC5313- and HLC54-infected mice; however, no particular species or genus, except the *Ruminococcaceae* family, was identified as a discriminative biomarker on day 12 with an LDA score ≥4 (**Figure 5D**). These results suggested that infection with both strains caused comparable gut microbiota alterations at the early stage, but the unique alterations associated with the different outcomes between SC5314 and HLC54 infections occurred at the late stage.

### The fecal fungal counts were positively correlated with the abundance of *Bacteroides vulgatus* and negatively correlated with the abundance of *Candidatus Arthromitus*

**Figure 6:**
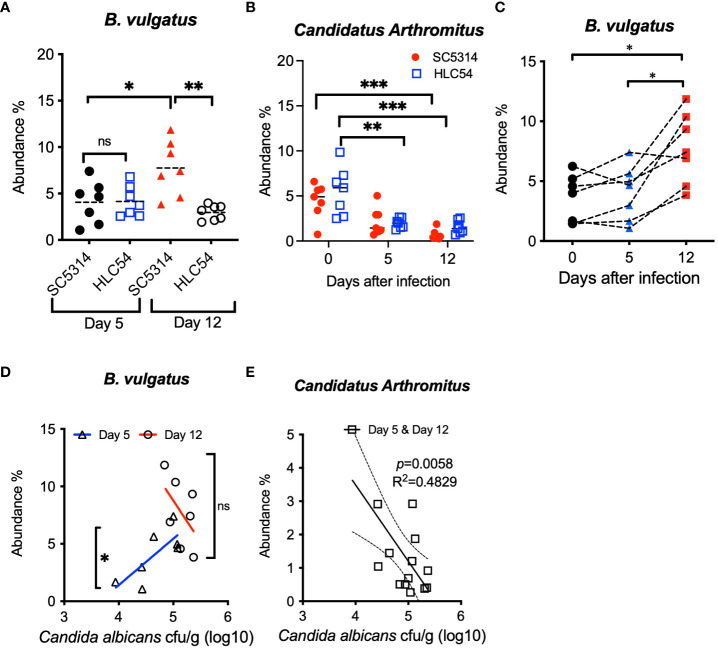
Comparison and correlation analysis of the differential abundances of fecal bacteria between SC5314- and HLC54-infected mice. Comparison of the *B. vulgatus* **(A)** and *Candidatus Arthromitus* **(B)** abundances between SC5314- and HLC54-infected Rag2γc mice before infection (day 0) and on days 5 and 12 post-infection is shown. For statistical analysis, 1-way ANOVA and 2-way ANOVA were used for *B. vulgatus* and *Candidatus Arthromitus*, respectively. Longitudinal changes in the fecal *B. vulgatus* abundance in individual Rag2γc mice infected with SC5314 at the indicated time points connected with a dashed line **(C)**. The relative abundances of *B vulgatus* **(D)** and *Candidatus Arthromitus* **(E)** and fecal fungal loads obtained from individual SC5314-infected mice at 5 and 12 days post-infection (n=7) were analyzed by linear regression, and the result is plotted at the best-fit line with 95% confidence bands. ("ns" indicates no significance, and the symbols *, ** and *** signify p values less than 0.05, 0.01 and 0.001, respectively).

In addition to the longitudinal analysis, we also compared the abundance of *B. vulgatus* and *Candidatus Arthromitus* between SC5314- and HLC54-infected mice to further confirm that the specific change in *B. vulgatus* abundance was associated with ICA. Only mice with disease onset (SC5314-infected mice at day 12) exhibited a significant increase in the abundance of *B. vulgatus*, compared to mice without the disease, including SC5314-infected mice on day 5 or HLC54-infected mice (**Figure 6A**). In contrast, the abundance of *Candidatus Arthromitus* displayed the same alteration between SC5314- and HLC54-infected mice, which consisted of a significant decrease on day 12 compared with the pre-infection levels (**Figure 6B**). To understand the consistency of the changes in *B. vulgatus* abundance among SC5314-infected mice, we traced the changes in *B. vulgatus* abundance over time in individual mice and found a significant increase in *B. vulgatus* abundance in SC5314-infected mice on day 12, and 6 out of 7 mice displayed an increasing trend in *B. vulgatus* abundance from day 5 to day 12 (**Figure 6C**). We further tested the dose correlation between fecal fungal counts and the abundance of *B. vulgatus* and *Candidatus Arthromitus*, two major discriminative biomarkers for *C. albicans* invasion and pre-infection. A positive correlation was found between fungal counts and *B. vulgatus* abundance early in SC5314 infection (day 5, *p*<0.05) but not in the later stage (day 12; **Figure 6D**). These results suggested that the enrichment of *B. vulgatus* was biphasic and included a fungal dose-dependent early increase in abundance followed by a further increase in abundance at later and fungal count-independent stages. In contrast, a significant negative correlation was observed between fungal counts and *Candidatus Arthromitus* abundance after SC5314 infection (combined results on days 5 and 12; n=14, *p*<0.01; **Figure 6E**). By combining the results from comparison and correlative analyses between SC5314- and HLC54-infected mice, *B. vulgatus* was the only species that served as a discriminative biomarker at the time of invasive infection onset.

### A correlated increase in IL-22 mRNA corresponding to the tissue fungal burden was observed in immunodeficient Rag2γc mice

**Figure 7:**
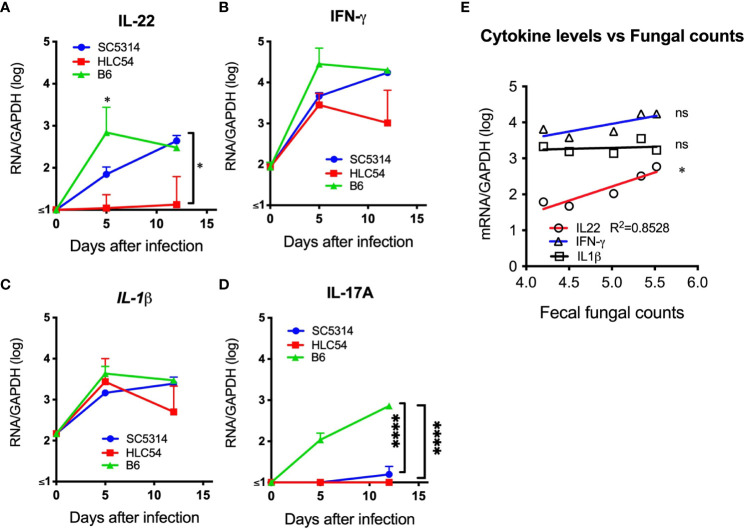
The levels of proinflammatory cytokine mRNA were higher in the stomach of *C albicans*-infected mice. The stomachs of immunodeficient Rag2γc mice (n=5) or immunocompetent C57BL/6 mice (n= 4) infected with SC5314 or HLC54 were harvested at the indicated time points, and the tissue mRNA levels were determined by quantitative RT−PCR and normalized to the mRNA levels of the housekeeping GAPDH gene. The means and standard deviations of the tissue IL-22 **(A)**, IFN-γ **(B)**, IL-1β **(C)** and IL-17A **(D)** mRNA levels are presented. Correlation analysis between the cytokine mRNA levels and tissue fungal loads of SC5314-infected Rag2γc mice was performed by linear regression, and the result is plotted as the best-fit line **(E)**. ("ns" indicates no significance, and the symbols * and **** signify p values less than 0.05 and 0.0001, respectively).

*C. albicans* can trigger host immune responses, particularly the production of inflammatory cytokines. To understand the cytokine responses after *C. albicans* infection, we quantified the levels of IL-22, IL-17A, IL-1β and IFN-γ RNA in stomach tissues from SC5314- and HLC54-infected immunodeficient Rag2γc mice. As a control, we also used SC5314-infected immunocompetent C57BL/6 mice, which displayed robust increases in the IL-22 and IFN-γ RNA levels that peaked on day 5 after SC5314 infection (**Figures 7A, B**). In contrast, SC5314-infected immunodeficient Rag2γc mice displayed delayed increases in the IL-22 and IFN-γ RNA levels, which peaked on day 12. HLC54-infected mice showed comparable IFN-γ RNA levels and significantly lower IL-22 RNA levels compared with SC5314-infected immunodeficient mice. No significant difference in the IL-1β response was found between the different groups (**Figure 7C**). These results suggested that neither infection with the fungal mutant nor immunodeficiency affected *C. albicans*-induced IL-1β responses in our model. Contrary to the rapid induction in immunocompetent C57BL/6 mice after SC5314 infection, IL-17A RNA expression was almost undetectable in immunodeficient Rag2γc mice (**Figure 7D**) due to the lack of IL-17A-producing lymphoid cells. According to the correlation analysis, only the IL-22 RNA levels, but not those of IFN-γ or IL-1β, were positively correlated with the fungal loads in tissues of SC5314-infected immunodeficient Rag2γc mice (**Figure 7E**). This result suggested that despite abnormal IFN-γ, IL-17A and IL-22 gene expression due to a delayed or no response to *C. albicans* in immunodeficient Rag2γc mice, the extent of the impairment varied between cytokines, and IL-22 appears to be less influenced among these cytokines.

## Discussion

Early initiation of antifungal therapy is associated with a reduced mortality of hospitalized patients with invasive candidiasis (Garey et al., 2006). In this study, we identified the enrichment of *B. vulgatus* in feces as a discriminative biomarker associated with ICA by comparing the differential changes in the gut bacterial composition between wild-type and filamentation-defective mutant *C. albicans-*infected mice. Our results provide an alternative strategy to solve the unmet problem of lacking rapid diagnostic biomarkers for ICA in immunocompromised individuals by measuring the abundance of particular fecal bacteria. Given that invasive candidiasis can be caused by non-albicans *Candida* spp. and occurred in immunocompetent hosts, our findings are still restricted to those invasive candidiasis cases with the immunocompromised underlying and caused by *C. albicans*.

The gut microbiota interacts with *C. albicans* in the GI tract and is probably the first microorganism to sense *C. albicans* switching from commensal to pathogenic. However, the use of antibiotics for *C. albicans* colonization in adult mice has impeded studies of the interplay between gut bacteria and *C. albicans.* The unique advantage of our immunodeficient Rag2γc mouse model is that it allows *C. albicans* to colonize and interact with gut bacteria in the absence of antibiotics. Of course, the deficiency of functional immune cells in this model also influences the gut microbiota composition. For example, compared with wild-type mice, Th2-deficient or Th1/Th2-deficient mice showed reductions in gut bacteria that produce short-chain fatty acids (SCFAs), including *Lactobacillus* spp., *Akkermansia muciniphila* and *Odoribacter* spp (Van Averbeke et al., 2022). In HIV-infected humans, a decrease in *Lactobacillales* abundance was found parallel to a reduction in the CD4 T-cell counts (Perez-Santiago et al., 2013). Considering the loss of functional intestinal IL-17- and IL-22-producing cells upon HIV/SIV infection (Ryan et al., 2016), our immunodeficient mouse model mimics HIV-infected patients with IL-17/IL-22 deficiency and an altered gut microbiota composition.

Among the gut microbiota, SCFA-producing bacteria are known probiotics for gut health and play an important role in inhibiting *C. albicans* colonization (Fan et al., 2015) and morphogenesis (Noverr and Huffnagle, 2004) and in suppressing *C. albicans-*induced mucosal inflammation (Bhaskaran et al., 2018). In agreement with other findings regarding GI infections caused by *C. albicans* (Mason et al., 2012b; Seelbinder et al., 2023) or other pathogens (Sohail et al., 2021), our data support the hypothesis that the introduction of *C. albicans* into the GI tract increased the gut bacterial alpha diversity and the abundance of SCFA-producing bacteria, especially butyrate-producing *Lachnospiraceae* and *Ruminococcaceae*, two abundant families in *Firmicutes* (Louis et al., 2014). Contrary to the blockade of *C. albicans* colonization observed in immunocompetent mice (Fan et al., 2015), the enrichment of *Lachnospiraceae* and *Ruminococcaceae* failed to prevent wild-type *C. albicans* colonization in Rag2γc mice. The ability to overcome colonization resistance seemed to be associated with hyphal formation because the colonization resistance was unaffected in filamentation-defective HLC54. Paradoxically, other reports have also indicated that SCFA-producing bacteria, particularly *Lactobacillus*, inhibit the germination of *C. albicans* in culture (Noverr and Huffnagle, 2004). Given that a reduced *Lactobacillus* abundance was detected in mice with genetic defects in IL-22 (Zenewicz et al., 2013) and because the basal level of IL-22 is reportedly much lower in Rag2γc mice (Satoh-Takayama et al., 2008), it is reasonable that a reduced abundance of *Lactobacillus* in immunodeficient Rag2γc mice attenuates the ability of the gut microbiota to resist *C. albicans* colonization. The findings that *C. albicans* enhanced the abundance of *Lachnospiraceae* and *Ruminococcaceae* and overcame the inhibition exerted by gut commensals in our immunodeficient mouse model are consistent with the observation in people with HIV (Gosalbes et al., 2022) and provide an explanation for the high frequency of candidiasis in HIV patients. Additionally, the observation that the fecal cultures of HLC54 mutant almost disappeared in Rag2γc mice suggests the protective role of the gut microbiota.

Despite the finding that *C. albicans* causes changes in the gut microbiota, the same bacterial alterations of *Clostridiaceae I*, *Lachnospiraceae* and *Ruminococcaceae* at the early stage (day 5) between SC5314- or HLC54-infected Rag2γc mice suggest that these are not differential changes between commensal and pathogenic *C. albicans*. Instead, our results indicated that the differential gut microbiota changes between SC5314- or HLC54-infected Rag2γc mice mainly occurred at the late stage, consistent with the time of disease onset. By longitudinal analysis and comparison between mice with invasive and noninvasive *C. albicans* infections, we identified the enrichment of *B. vulgatus* in feces as the major discriminative biomarker associated with ICA and found a positive correlation between the *B. vulgatus* abundance and fecal fungal load early in infection. This study provides the first *in vivo* evidence supporting the correlation between *B. vulgatus* and *C. albicans*, although *in vitro* coculture results have previously demonstrated that *C. albicans* significantly enhances the growth of *B. vulgatus* and *B. fragilis* via oxygen consumption and nutrient supplementation (Valentine et al., 2019). *B. vulgatus* together with *B. fragilis* are also responsible for a variety of infections that cause bacteremia or abscess formation in multiple body sites (Wexler, 2007). Interestingly, in the case of inflammatory bowel disease (IBD) patients, both *C. albicans* and *B. vulgatus* have been linked to worse outcomes because positive associations for the *B. vulgatus* protease levels with disease severity (Mills et al., 2022) and aggravation of intestinal inflammation by *C. albicans*-secreted candidalysin (Li et al., 2022) have been found. Whether *C. albicans* and *B. vulgatus* cooperatively contribute to ICA pathogenesis remains unclear; however, *in vitro* cocultivation indicates that *B. vulgatus*, but not *B. fragilis*, promotes *C. albicans* growth in a fungal strain-specific manner (Valentine et al., 2019). Conversely, *B. thetaiotaomicron* has been reported to closely associate with *C. albicans* yeast within GI mucus (Eckstein et al., 2020) and inhibit *C. albicans* colonization in the mouse gut (Fan et al., 2015), and the secreted metabolites from *B. ovatus* display antifungal activity in culture (Garcia et al., 2017), suggesting an inhibitory effect of *Bacteroides* on *C. albicans.* These evidences suggest that *Bacteroides* interacts with *C. albicans* in the gut and that the interactions between *Bacteroides* and *C. albicans* differ among *Bacteroides* spp.

In addition to *Bacteroides*, the existence of *C. albicans* in the cefoperazone-treated mouse gut has been reported to facilitate the rehabilitation of *Enterococcus* spp. in postantibiotic recovery (Mason et al., 2012a). However, in our mouse model, the *Enterococcus* abundance was not changed because SCFA-producing bacteria, which are suppressed in antibiotic-treated mice, were highly abundant and inhibited the growth of *Enterococcus* spp (Jeong et al., 2019).

In addition to the cross-kingdom interplay between *C. albicans* and the gut microbiota, *C. albicans* commensalism is also under immunosurveillance by epithelial cells and innate immune cells, both of which recognize *C. albicans* via various pathogen-recognition receptors and initiate host defenses, including nonspecific innate immunity and specific adaptive immunity (d'Enfert et al., 2021). Reportedly, epithelial cells produce proinflammatory cytokines, such as IL-1β, IL-6, IL-8 and TNF-α, in response to *C. albicans* infection (Villar et al., 2005). Consistently, the comparable increase in IL-1β RNA transcription in both immunodeficient and immunocompetent mice indicated no defects in *C. albicans*-triggered IL-1β production in Rag2γc mice. Contrary to the unaffected increase in the IL-1β levels, the levels of cytokines associated with predominant antifungal T-cell responses, including IFN-γ, IL-17A and IL-22 (Curtis and Way, 2009; De Luca et al., 2010), were largely reduced in Rag2γc mice due to the deficiency of functional lymphoid cells. Among these cytokines, only the IL-22 mRNA levels differed between SC5314- and HLC54-infected mice. In addition, a positive correlation between the IL-22 mRNA levels and fungal loads was found in SC5314-infected Rag2γc mice. This result implicated the different extents of impaired responses among IFN-γ, IL-17A and IL-22 cytokines, and IL-22 is still responsible for *C. albicans* infection, even with a delayed increase and an inability to prevent *C. albicans* colonization.

In fact, a surge in the production of antimicrobial peptides and mucins driven by IL-22 not only promotes defense against fungal infection but also shapes the microbiota composition (Dudakov et al., 2015). For example, the segmented filamentous bacterium (SFB) abundance was observed to be higher in IL-22-deficient and Rag2γc mice, and the administration of anti-IL-22-neutralizing antibody or IL-22-Fc fusion protein could modulate the SFB abundance in the gut (Shih et al., 2014). In line with this finding, we found that the abundance of clostridial *Candidatus Arthromitus* (occasionally designated *Candidatus Savagella*), a candidate SFB species (Hedblom et al., 2018), was decreased in Rag2γc mice after exposure to *C. albicans* infection and negatively correlated with the fungal load, possibly mediated by elevated IL-22 levels. This finding was not clearly seen in other studies investigating the recovery of the gut microbiota after antibiotic treatment because *Candidatus Arthromitus* is susceptible to penicillin or vancomycin used in those studies (Fan et al., 2015; Shankar et al., 2015). SFB species have been reported to be potent inducers of Th17 differentiation in the lamina propria (Ivanov et al., 2009; Roy et al., 2021). Although *C. albicans* can induce the production of specific Th17 cells mainly via fungal cell wall components (Hernandez-Santos and Gaffen, 2012), the reduction in *Candidatus Arthromitus* abundance induced by *C. albicans* indicated that *Candida* and other commensal fungi might serve as potent stimulators to elevate the basal level of IL-22 and downregulate the differentiation of host intestinal Th17 cells, which are reportedly involved in allergic airway diseases (Noverr et al., 2004) and autoimmune disorders (Bradley et al., 2017).

It is hypothesized that the tripartite interaction among host immunity, the microbiota and *C. albicans* coordinately regulates the commensalism and pathogenesis of *C. albicans* in the gut. Our results revealed that in the presence of the altered gut microbiota in immunocompromised hosts, *C. albicans* was able to colonize and invade the GI mucosa and promote the abundance of *B. vulgatus*. Of course, the application of rapid diagnosis for ICA by measuring the abundance of *B. vulgatus* in feces is fantastic, but an evaluation in clinical patients is still needed. Notably, *B. vulgatus* has also been reported as an associated biomarker of IBD (Mills et al., 2022), type-2 diabetes (Bakir-Gungor et al., 2021) and cancer (Usyk et al., 2021; Teng et al., 2023); therefore, the role of *C. albicans* in these diseases needs to be carefully investigated. Recently, the transplantation of probiotic bacteria or biotherapy has been proposed to inhibit the overgrowth of pathogenic *C. albicans* in the gut. Our current immunodeficient mouse model can fit the clinical settings of immunocompromised patients and be used to evaluate the efficacy of biotherapy.

## Materials and methods

### Ethics statement

C57BL/6 female mice aged 6-8 weeks obtained from the National Laboratory Animal Center (Taipei, Taiwan) and 6- to 8-week-old Rag2^-/-^IL2γc^-/-^ (Rag2γc) male and female mice originating from Taconic Farms (Bar Harbor, Maine USA) and bred by our research group under authorization were maintained in the animal facility of the National Health Research Institutes. The protocol was approved by the Animal Committee of the National Health Research Institutes (protocol No: NHRI-IACUC-103013-A) and performed according to their guidelines. All mice were housed in ventilated cages in a specific pathogen-free environment and supplied sterile bedding. Water and food were given *ad libitum*.

### *Candida albicans* strains and culture conditions

*Candida albicans* wild-type SC5314 and its yeast-restricted mutant HLC54 (*cph1/cph1 efg1/efg1*) were used in this study. The strains were stored in vials at -70°C and plated on Sabouraud dextrose agar (SDA) for overnight growth. The refreshed colonies were continually grown in yeast extract-peptone-dextrose (YPD) broth at 37°C overnight, washed and resuspended in PBS. The concentration of *Candida albicans* was determined by a hemocytometer.

### Murine model of GI colonization and disseminated candidemia induced by *C. albicans*

For infection, 6- to 8-week-old Rag2γc (n=7) or C57BL/6 mice (n=4) were orally injected with 1 x 10^7^ *Candida albicans* by gavage (FTP-20-38, Instech Laboratories, PA, USA). The body weight was monitored at particular time points unless the mice became moribund (defined as a decrease of over 20% of the initial body weight or inability to self-feed). Stool was collected from individual mice at particular time points, weighed and homogenized in 0.5 ml of PBS. Then, 200 μl of serial 10-fold dilutions of the homogenates were plated on YPD plates containing chloramphenicol (62.5 μg/ml; Sigma, St. Louis, MO, USA) and incubated at 30°C for two days. The number of fungal colonies was counted and normalized to the sample weight.

### Tissue staining

Tissue specimens were obtained from *C. albicans*-infected mice, rinsed with PBS and fixed with formalin. After being embedded in paraffin, the tissue blocks were sectioned and stained with periodic acid-Schiff (PAS) staining with hematoxylin for counterstaining.

### Gut microbiota 16S rRNA gene sequence analysis

Stool samples collected from individual mice were stored at -20°C until use. Fecal DNA was purified using a DNA isolation kit (MP Bio, Solon, OH, USA) based on the manufacturer’s instructions within one week after the end of the experiments. The composition of the gut microbiota was analyzed by next-generation sequencing of the bacterial 16S rRNA gene. In brief, a 16S rRNA sequencing library was constructed according to the metagenomic sequencing library preparation protocol (Illumina, San Diego, CA, USA) targeting the V3 and V4 hypervariable regions of the 16S rRNA gene (341F-805R) using specific primers (forward primer: 5′-TCG TCG GCA GCG TCA GAT GTG TAT AAG AGA CAG TCG TCG GCA GCG TCA GAT GTG TAT AAG AGA CAG CCT ACG GGN GGC WGC AG-3′; reverse primer: 5′-GTC TCG TGG GCT CGG AGA TGT GTA TAA GAG ACA GGT CTC GTG GGC TCG GAG ATG TGT ATA AGA GAC AGG ACT ACH VGG GTA TCT AAT CC-3′). Subsequently, the purified PCR products were quantified with a Qubit 3.0 fluorometer (Thermo Fisher Scientific, Carlsbad, CA, USA). The pooled samples were run on an Agilent 2200 Tape Station (Agilent Technologies, Santa Clara, CA, USA) for quality analysis prior to sequencing. The samples were prepared following Illumina guidelines and sequenced on the MiSeq sequencing platform according to the standard Illumina sequencing protocols. The 16S rRNA gene sequence data were analyzed by QIIME (version 1.9.0) (Caporaso et al., 2010). The raw data were cleaned to remove the sequences of adapters, primers and low-quality bases, assembled into reads by FLASH (version 1.2.11) and clustered by the UCHIME algorithm. The quality-filtered and nonchimeric reads were then analyzed to generate operational taxonomic units (OTUs) for each sample based on 97% similarity. The SILVA 132 OTU collection was used for bacterial OTU taxonomy assignment (Quast et al., 2013).

### Quantitative RT−PCR

For quantitative RT−PCR, the total RNA isolated from tissue homogenates using TRIzol (Invitrogen, Carlsbad, CA, USA) and an RNA clearance kit (Qiagen, Hilden Germany) was reverse transcribed to cDNA using Superscript III (Invitrogen) and stored at -80°C until use. The levels of IL-17A, IL-22, IFN-γ and IL-1β cDNA were determined by quantitative PCR (LightCycler ® 480, Roche, Rotkreuz, Switzerland) with the TaqMan primer and the probe sets listed in the **Supplementary Material** (**Table S1**) and normalized to the cDNA levels of the housekeeping gene GAPDH.

### Statistical analyses

Alpha diversity measurements and principal coordinate analysis were performed using QIIME. Statistical analysis of metagenomic profiles was performed by STAMP version 2.1.3 (Parks et al., 2014). Multigroup and two-group analyses were performed by the Kruskal−Wallis *H* test and Mann−Whitney test using STAMP. Differentially abundant taxa were identified using linear discriminant analysis (LDA) effect size (LEfSe) methods (Segata et al., 2011). Other statistical analyses were performed by 2-way ANOVA with the Bonferroni posttest (GraphPad Prism), unless otherwise specified. Correlations between two proportions were determined by the Spearman method. Differences with a *p* value less than 0.05 were considered statistically significant.

## Data availability statement

The datasets presented in this study can be found in online repositories. The names of the repository/repositories and accession number(s) can be found below: https://figshare.com/articles/dataset/23813448.

## Ethics statement

The animal study was approved by IACUC National Health Research Institutes, Taiwan. The study was conducted in accordance with the local legislation and institutional requirements.

## Author contributions

J-YY: Formal analysis, Methodology, Writing – review & editing, Investigation, Project administration. T-HL: Investigation, Writing – review & editing. Y-TJ: Investigation, Writing – review & editing, Formal analysis. J-WH: Investigation, Writing – review & editing. S-HW: Investigation, Writing – review & editing. H-JL: Writing – review & editing. Methodology. Y-CC: Writing – review & editing, Conceptualization. C-HP: Conceptualization, Writing – review & editing, Formal analysis, Funding acquisition, Methodology, Software, Supervision, Writing – original draft.
